# Bariatric Surgery and Its Metabolic Echo Effect on Serum Uric Acid Levels

**DOI:** 10.7759/cureus.58103

**Published:** 2024-04-12

**Authors:** Subodh Bashyal, Shen Qu, Manoj Karki

**Affiliations:** 1 Department of Endocrinology and Metabolism, Shanghai Tenth People's Hospital, School of Medicine, Tongji University, Shanghai, CHN; 2 Shanghai Center of Thyroid Diseases, Shanghai Tenth People's Hospital, School of Medicine, Tongji University, Shanghai, CHN; 3 SinoUnited Health, Endocrinology, Metabolism and Thyroid Center, Shanghai, CHN; 4 Department of Internal Medicine, Endocrinology and Metabolism, Universal College of Medical Sciences, Tribhuvan University, Bhairahawa, NPL

**Keywords:** gout crystals, metabolic syndrome (mets), hyperuricemia, serum uric acid, bariatric surgery

## Abstract

Bariatric surgery (BS) has been a significant means of reducing weight in obese individuals. The metabolic changes after bariatric surgery are crucial as they extend its advantages beyond weight loss. As its name implies, “metabolic surgery” also addresses obesity-related metabolic concerns. Bariatric surgery has always been associated with lessened serum uric acid (SUA) levels. In this review, we examined current studies to understand how surgical therapies impact serum uric acid levels. Strongly minded on the extent and timing of changes in the level of serum uric acid after bariatric surgeries. We conducted a comprehensive search for relevant current studies in PubMed, Google Scholar, JAMA, and the Cochrane Library until February 1, 2024. We aimed to analyze the metabolic advantages of bariatric surgery, focusing on its function in treating hyperuricemia and lowering the risk of associated disorders. Our review elaborates on factors contributing to decreased serum uric acid levels after bariatric surgery, such as alterations in renal function, insulin sensitivity, and inflammatory markers.

## Introduction and background

Obesity is a complex disease condition characterized by the deposition of excessive amounts of body fat [1]. In this era of fast food and sedentary lifestyles, obesity is a well-established form of malnutrition around the globe [2]. Recent figures from the World Health Organization (WHO) show that 2.5 billion individuals aged 18 years and older are overweight, with more than 890 million people classified as obese. 16% of the global adult population was obese in 2022. Additionally, 43% were overweight, highlighting the concerning impact of obesity as an epidemic. There was not much gender disparity, with 43% of men and 44% of women being overweight in the total population [3]. It further reports that 2.8 million people die each year owing to obesity and overweight [4]. Obesity, being a chronic disease, is also linked with many other disease conditions. Obesity is associated with various disease conditions related to the heart, kidney, musculoskeletal system, cancer, psychological disorders, and metabolic disorders [5]. Among these parameters, many studies have reported on the link between serum uric acid (SUA) and body weight gain [6]. Some recent studies have reported that individuals with hyperuricemia have a significantly higher body mass index (BMI) [7,8]. After a two-year follow-up of 3,153 individuals, Ishizaka et al. reported BMI change was a main factor for SUA decrement [9]. As the association between obesity and SUA is well established, weight is an important modifiable risk factor for hyperuricemia. Obesity has several other impacts that range beyond physical health, including substantial psychological consequences like social isolation, social stigma, and depression [10]. Being concerned about these demerits of obesity, clinicians are using various weight reduction methods. The methods of weight reduction range from lifestyle modification to weight reduction drugs and sophisticated bariatric surgery (BS) [11]. Bariatric surgeries are more considered these days over other treatment protocols due to their role in long-term weight loss in severely obese individuals [12]. It has been established that BS has a tangible role not merely in weight loss but in improving various other metabolic health parameters. Recent studies indicate that BS can significantly decrease uric acid levels. This fall in SUA helps to lower the risk of gout and other urate-related illnesses [13]. Studies show a varied range of results, which may be due to the type of BS used, the extent of weight loss, and other conditions like renal dysfunction [14]. This review intends to analyze the current findings about the change in uric acid levels after BS. It also strives to clarify the factors responsible for the extent and timing of the falls in uric acid after BS by coalescing results from various studies.

## Review

Obesity, bariatric surgery types, and indications

Obesity is characterized by the deposition of excessive amounts of fatty tissue in the body. The WHO classifies obesity into different categories based on BMI levels: overweight is defined as a BMI of 25 kg/m^2^ or higher, obesity is characterized by a BMI of 30 kg/m^2^ or higher, and severe obesity is described as a BMI greater than 40 kg/m^2^ [15]. Various other metrics, such as waist circumference (WC), waist-to-hip ratio (WHR), body fat percentage (BFP), lean body mass (LBM), and visceral fat rating (VFR), are used to define obesity and body fat distribution [16]. Obesity's causation is complex, involving genetic predisposition, cultural behaviors, socioeconomic level, and environmental factors [17]. The complexity of factors responsible for obesity indicates the necessity of using a comprehensive strategy for obesity management.

BS is a highly successful surgical therapy for severe obesity or obesity associated with other comorbid medical conditions [11]. The term "bariatric" originates from the Greek terms "baros," referring to weight, and "iatriki," which means medicine [18]. Bariatric surgeries are mainly indicated for individuals with a BMI above 40 kg/m^2^ or for those with a BMI of 35 kg/m^2^ or more with obesity-related comorbidities such as type 2 diabetes, hypertension, or severe sleep apnea [19]. Besides BMI and comorbidities, other factors, such as general health conditions and previous unsuccessful weight loss attempts with other methods, should also be considered [20]. Advancements in BS have been marked by significant improvements in surgical techniques over time. BS works on the mechanisms of limiting food intake, reducing nutrient absorption, and a combination of both [20]. Procedures such as laparoscopic adjustable gastric band (LAGB) and sleeve gastrectomy (SG) target decreasing stomach capacity to control the amount of food consumed. Biliopancreatic diversion (BPD) aims to decrease nutrition absorption by changing the anatomy of the digestive tract [21]. The Roux-en-Y Gastric Bypass (RYGB) procedure involves constructing a small stomach pouch by resection and redirecting food directly to the small intestine. It helps to reduce food intake and improve hormonal responses that promote weight reduction and feelings of early satiety during meals [22]. The SG is known for its long-term effectiveness in weight loss. The long-term effect of SG is believed to be due to the removal of a large part of the stomach, subsequently lowering ghrelin hormone levels and decreasing appetite [23]. Adjustable gastric banding (AGB) is a minimally invasive procedure that enables an alterable quantity of food intake [24]. The biliopancreatic diversion with duodenal switch (BPD/DS) and the single anastomosis duodeno-ileal bypass with sleeve gastrectomy (SADI-S) are also combined procedures that include both stomach reduction and intestine rerouting to reduce absorption and promote weight loss [25]. Although highly useful for weight loss and improving comorbidities, patients with bariatric surgeries require lifetime dietary alterations. Sometimes, dietary supplements to correct the nutritional deficits are required [26].

Uric acid, hyperuricemia, and its relationship with obesity 

**Figure 1:**
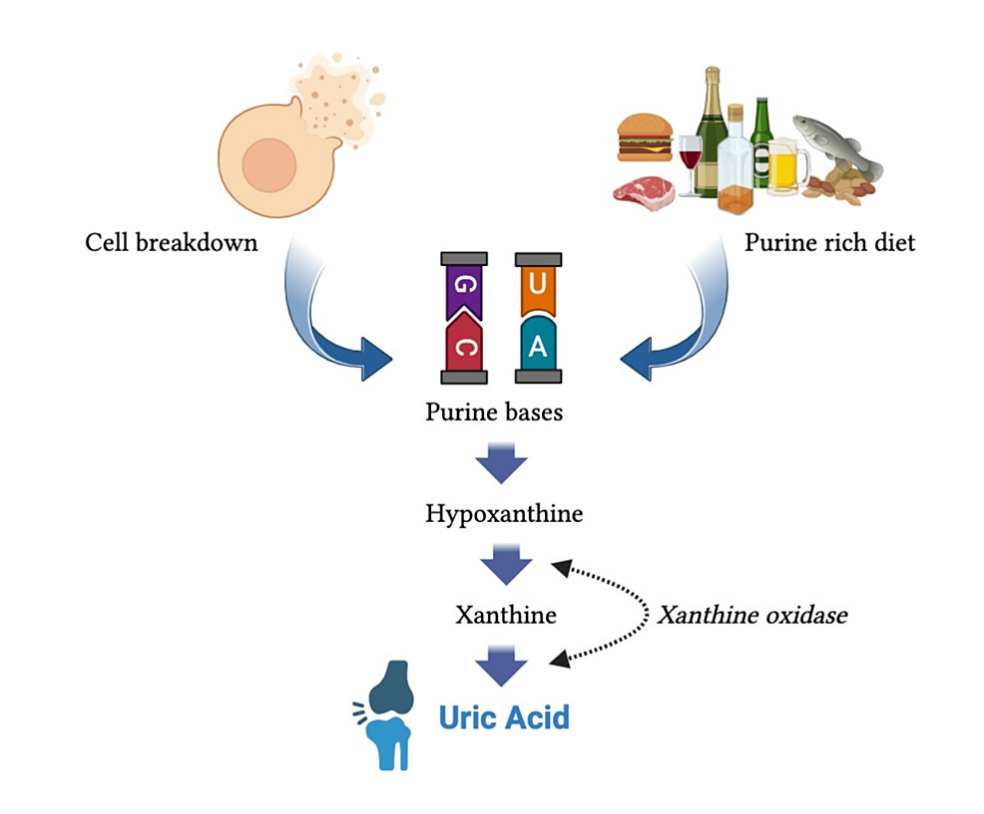
Demonstration of uric acid formation pathway; excessive purine-rich diet and cell breakdown leads to hyperuricemia. This image is created by Subodh Bashyal.

Uric acid is an end product of purine metabolism in the body. Ingestion of foods containing high levels of purines is primarily responsible for the high uric acid level in the blood. Foods with high levels of purine include red meat, seafood, beans, and alcohol products. Besides this, in vitro production of the purines during cell breakdown is another source of SUA (Figure 1). Conditions such as hemolysis and tumor lysis can raise uric acid levels, boosting cell turnover [27,28]. Then, this uric acid, due to the lack of uricase enzyme in humans, accumulates in the blood. Uricase is an enzyme of purine metabolism that changes uric acid to allantoin [29]. The kidneys can readily eliminate allantoin, which is a more hydrophilic molecule. The absence of uricase aids in the accumulation of uric acid in the body, as it is hard for the kidney to eliminate uric acid in abundance. This may lead to conditions such as hyperuricemia and, eventually, gout [30]. Since more than 70% of uric acid is eliminated by the kidneys, along with the greater production in the body, the inability of the kidney due to renal dysfunction or a combination of both can lead to hyperuricemia [31]. Hyperuricemia is identified by high amounts of uric acid in the blood, generally considered to be more than 7.0 mg/dL (416 µmol/L) for men and greater than 6.0 mg/dL (357 µmol/L) for women [32]. Hyperuricemia has a diverse epidemiology, and it is affected by food habits, genetic predisposition, and various underlying medical conditions. The higher prevalence of hyperuricemia is usually reported in the urban areas of different countries [33,34]. The high prevalence of hyperuricemia and gout in Pacific Islanders strongly supports the notion of feeding habits and genetics as causes of hyperuricemia [35]. Hyperuricemia is associated with various health conditions, such as obesity, metabolic syndrome, diabetes mellitus, cardiovascular diseases (CVDs), hypertension, and chronic kidney disease [36]. Usually, hyperuricemia may remain a silent disease and only be diagnosed in an instance of gout flare or nephrolithiasis [37].

Recent studies have noted a significant relationship between hyperuricemia and obesity. Hyperuricemia is found to be correlated with various phenotypes of obesity, such as metabolically healthy obese (MHO), metabolically abnormal non-weight (MANW), and metabolically abnormal obese (MAOO). There was an increasing trend of SUA in all the phenotypes [38]. Another similar study found a higher risk of hyperuricemia in metabolically unhealthy obese individuals than in metabolically healthy non-obese individuals [39]. A study from National Health and Nutrition Examination Survey (NHANES) data shows that food habits directly influence hyperuricemia, with obesity playing an important mediating role in this connection. A higher BMI was associated with a higher level of SUA in individuals with obesity. This study also indicated that the chance of hyperuricemia in obese people was significantly associated with insulin resistance [40]. Hyperuricemia is also commonly linked to a higher BMI, hypertriglyceridemia, and hypertension [41].

Post-bariatric surgery SUA level

BS is an essential procedure for obese individuals, offering a method for sustainable weight loss and addressing several metabolic issues, including hyperuricemia [42]. Following BS, patients often have a significant decrease in uric acid levels in the long term, reducing the risk of developing gout and related conditions. This change often happens in the first few months after surgery and lasts for a prolonged duration. Studies have demonstrated a strong association between the decrease in uric acid levels and the extent of weight loss, highlighting the direct impact of weight reduction on uric acid metabolism (Table 1). A thorough evaluation and meta-analysis have shown consistent decreases in uric acid levels after several bariatric procedures, improving gout outcomes and related symptoms. The study highlights the necessity of doing extended follow-up evaluations to determine the lasting effects of the metabolic benefits and the potential need for lifestyle and medication adjustments post-surgery [13].

**Table 1:** Trends in SUA levels after bariatric surgery in obese individuals BS: bariatric surgery, LSG: laparoscopic sleeve gastrectomy, RYGB: Roux-en-Y gastric bypass, SG: sleeve gastrectomy, SUA: serum uric acid, T2DM: type 2 diabetes mellitus. This table was created by Subodh Bashyal.

Year of the study	Sample size	Follow-up duration	Findings (SUA)	Author
2019	147	1, 3, 6, 12 (months)	Fall in the level than baseline in every follow-up in the patients with T2DM	Liu et al. [47]
2021	147	7 (days), 1, 3, 6, 12	A rapid decrease in SUA levels was observed after 7 days, followed by an increase at the 1-month follow-up. Subsequent follow-ups showed a significant reduction in SUA levels, particularly pronounced in patients previously diagnosed with hyperuricemia.	Lu et al. [48]
2011	420	8 (month)	Subsequent weight reduction following RYGB can decrease uric acid levels and the occurrence of hyperuricemia in severely obese individuals.	Serpa Neto et al. [49]
2015	820	3, 12, 24, 36 (months)	Uric acid levels decreased from 0.49 ± 0.06 mmol/l to 0.41 ± 0.05 mmol/l in three months and further to 0.3 ± 0.03 mmol/l over three years, indicating the BS's sustained positive effect on reducing hyperuricemia.	Van Der Merwe et al. [45]
2015	55	1 (month)	The mean SUA rose in the 1-month follow-up, contrary to the anticipated decrease. This unexpected increase indicates short-term postoperative influences on uric acid levels.	Menenakos et al. [50]
2013	60	2 weeks, 1, 3, 6, 12 (months)	Uric acid levels rise to 0.44 mmol/L immediately after surgery at 2 weeks. At 3 months, the level begin to drop. It continues to fall after 6 and 9 months, and by 12 month it drops significantly to 0.30 mmol/L.	Dalbeth et al. [44]
2018	85	1 (month)	Significant reduction in the prevalence of hyperuricemia from 30.6% pre-operatively to 18.8% post-operatively following LSG.	Katsogridaki et al. [51]
2023	41	3, 6, 12 (months)	Significant decrease in SUA levels at 6 and 12 months post-surgery in severely obese patients.	Vafa et al. [42]
2020	252	12 (month)	The baseline SUA levels were 6.6 ± 1.7 mg/dL, and at the 12-month follow-up, it decreased to 5.7 ± 1.5 mg/dL (P = 0.001). Indicated a positive impact of BS on reducing SUA levels among patients without renal function impairment. Top of Form.	Hung et al. [14]
2021	34	1 (week), 3, 6 (months)	Serum urate levels showed a significant increase in 1 week but decline was observed at 1 month and 3 months follow up post-surgery.	Li et al. [52]
2018	128	6 (month)	SUA levels dropped significantly at six months after surgery, especially in individuals with higher pre-surgical levels. The degree of decrement was greater in women than men.	Zhang et al. [53]
2015	100	3,6,12(months)	The study demonstrates a consistent and significant decrease in uric acid levels across all obesity groups (mild, moderate, and severe) at 3 and 6 months follow-up after BS.	Chawla et al. [54]
2022	98	3, 6 (months)	SUA levels dropped considerably after 3 months and continued for 6 months after surgery. SUA levels dropped significantly from 5.56 mg/dl pre-surgery to 4.32 mg/dl 6 months post-surgery.	Balata et al. [55]
2017	3981	6 (months) and 1, 2, 3, 4, 6, 8, 10, 15 and 20 (years)	After excluding baseline hyperuricemia people, 314 control group subjects acquired hyperuricemia during follow-up, compared to 188 surgical group subjects. This shows that BS reduces uric acid levels over 20 years.	Maglio et al. [56]
2018	40	12 (month)	At 12 months follow up post-surgery, the low-purine diet group showed a significant reduction in blood uric acid levels compared to the normal-purine diet group after SG (p < 0.001).	Schiavo et al. [57]
2022	36	3, 6, 12(months)	After 12 months after sleeve gastrectomy, male and female uric acid levels dropped from 8.6 to 6.3 mg and 8.0 to 5.6 mg, respectively, with statistical significance (p = 0.003).	Alwadaani [58]

Comparing various BS procedures, GB and SG had a more significant effect on uric acid levels than adjustable gastric banding. The variations are likely a result of the varying degrees of weight reduction and hormonal alterations induced by each surgery method [43]. Initial post-surgical findings usually indicate a rapid increase in uric acid levels within the first few days to weeks. The rapid metabolic changes in the body, tissue breakdown, and initial renal adaptation are believed to be responsible for this transient increase in uric acid production [44]. Over the months and years, it has been seen that the continuous decrease in uric acid levels is maintained, along with sustained weight loss [45]. This lasting impact is crucial for preventing chronic diseases, especially by lowering the likelihood of developing gout and kidney stones [46].

The factors responsible for the decrement of SUA after bariatric surgery

**Figure 2:**
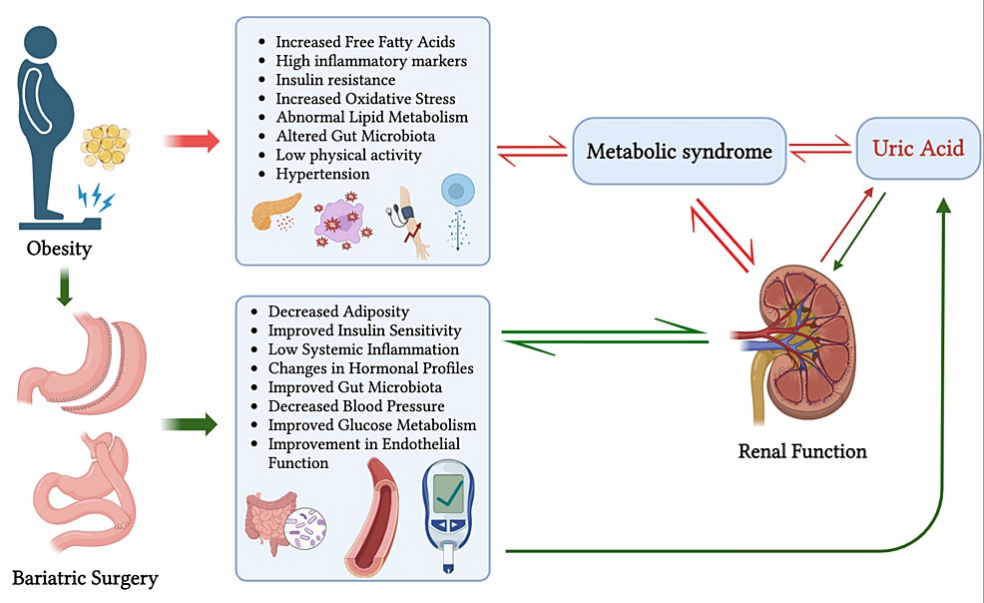
The influence of pre-bariatric surgery metabolic syndrome and post-bariatric surgery metabolic improvements in the determination of serum uric acid level. This image is created by Subodh Bashyal.

Many studies indicate an early rise in the SUA level, followed by a decrease in subsequent follow-ups [44]. This is believed to be due to the rapid weight loss, changes in renal function, and altered purine metabolism in the body. Post-BS, there is generally rapid and substantial weight loss. This rapid weight loss leads to fat cell breakdown for energy. The purines in these adipose cells are released and cause a surge of uric acid in the blood. The surgical trauma, change in blood volume, or fluid imbalance after the surgery affect kidney function. This alteration in the kidney’s function is another factor in the early rise in uric acid after BS [52,59]. There are multiple factors responsible for the long-term decrement in SUA levels. The insulin resistance resulting from increased insulin in the bloodstream hinders renal function [60,61]. Following BS, the enhancement in insulin sensitivity aids in improving renal function and concurrently causes a sufficient renal clearance of uric acid [62,63]. The enhancement in blood pressure control and improved cardiovascular health following weight loss have a role in renal function improvement and the elimination of uric acid [64]. The alteration of gut hormones responsible for appetite and glucose metabolism, such as the GLP-1 hormone, also aids in improving insulin sensitivity and secretion [65]. The alteration of the gut microbiota after the surgery is linked to intestinal uric acid clearance [48]. The adipose tissue not only acts as fat storage, but it also has a notable endocrine function. Adipose tissue in the obese undergoes cellular and molecular changes. This results in the release of cytokines and adipokines, which have pro-inflammatory and anti-inflammatory properties. Tumor necrosis factor-alpha (TNF-α), interleukin-6 (IL-6), and monocyte chemoattractant protein-1 (MCP-1) are some cytokines released from adipose tissue. These cytokines lead to widespread inflammation and are associated with the causation of insulin resistance, CVD, and other obesity-related comorbidities. This rise in inflammatory markers is directly or indirectly associated with the rise in uric acid. TNF-α and IL-6 have been found to affect renal blood flow and glomerular filtration primarily. These cytokines, by various means, such as increasing insulin resistance, disturbing cardiovascular health, increasing cell turnover, endothelial dysfunction, and renal dysfunction, eventually surge the SUA level in obese individuals [66,67]. BS results in sturdy weight loss, resulting in a reduction in the secretion of inflammatory cytokines [68]. This, in due course, reduces the generation of uric acid and lifts its elimination due to improved renal function (Figure 2). Changes in dietary habits after BS and the inability of the intestine to absorb all nutrients, including fructose and purines, due to intestinal bypasses lead to a decrease in uric acid levels [57]. Hormonal changes, such as a reduction in leptin resistance and an elevation of adiponectin, also play a role in the fall of SUA following BS. These hormonal changes are also associated with a decrease in inflammatory reactions, an improvement in insulin sensitivity, blood pressure control, and renal function improvement [69,70].

## Conclusions

Our review highlights the consistent finding of decreased SUA levels after surgery, as in the previous studies. However, the factors related to a decrease in SUA levels have been scarcely discussed at times. Our review suggests that this decrease in the level of SUA is caused by a combination of many processes. Improvement of renal function, increased insulin sensitivity, and changes in inflammatory markers after BS were some of the inescapable factors. After surgery, there may be a temporary rise in SUA levels due to rapid weight loss and metabolic changes due to surgical trauma. However, long-term follow-ups typically reveal a notable and consistent reduction in SUA levels. Thus, these findings suggest that the BS for weight loss leads to a significant reduction in blood uric acid levels in the long run in obese and severely obese individuals. BS has a significant role in managing pre-surgery uncontrolled hyperuricemia and repeated attacks of gout, along with the primary target of weight loss.
